# Supplementary figures and images for: Effects of Long-Term Physical Activity and BCAA Availability on the Subcellular Associations between Intramyocellular Lipids, Perilipins and PGC-1α
Source: Int J Mol Sci. 2023 Feb 21;24(5):4282. doi: 10.3390/ijms24054282 (PMC10002284; doi:10.3390/ijms24054282)

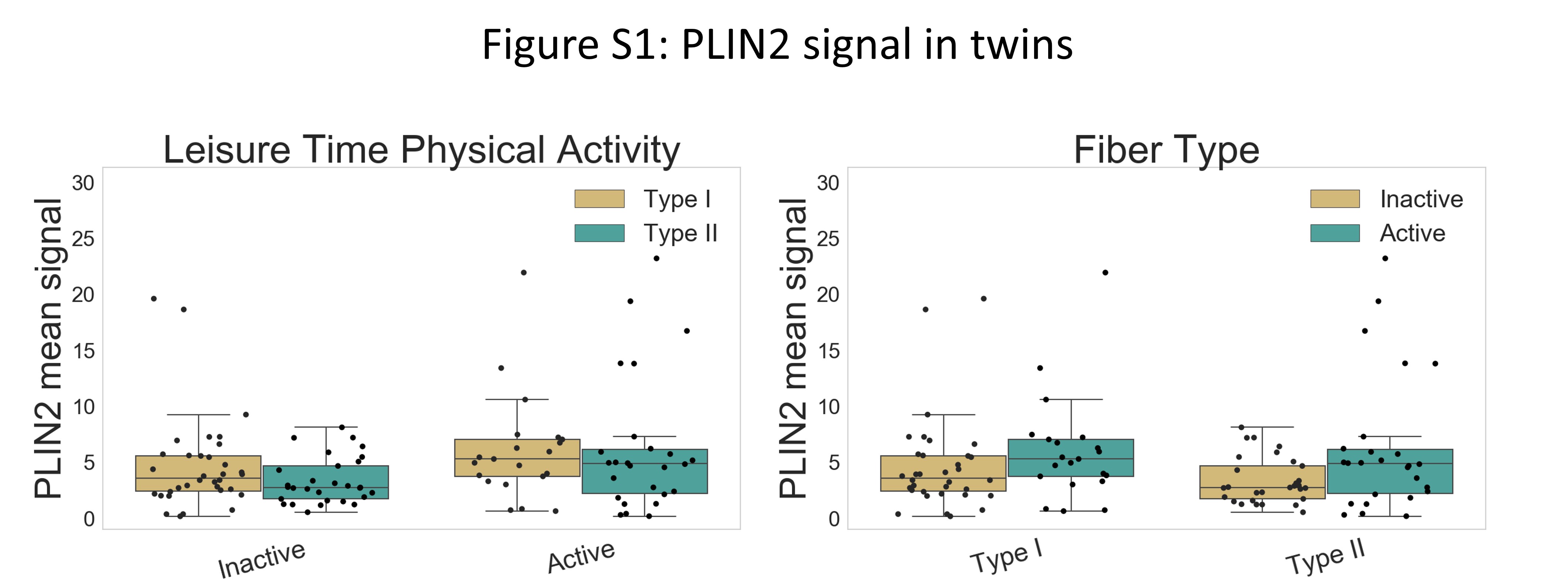

Supplement: Supplementary file 1 [file ijms-24-04282-s001.zip › supp_Updated/Sup1.jpg]

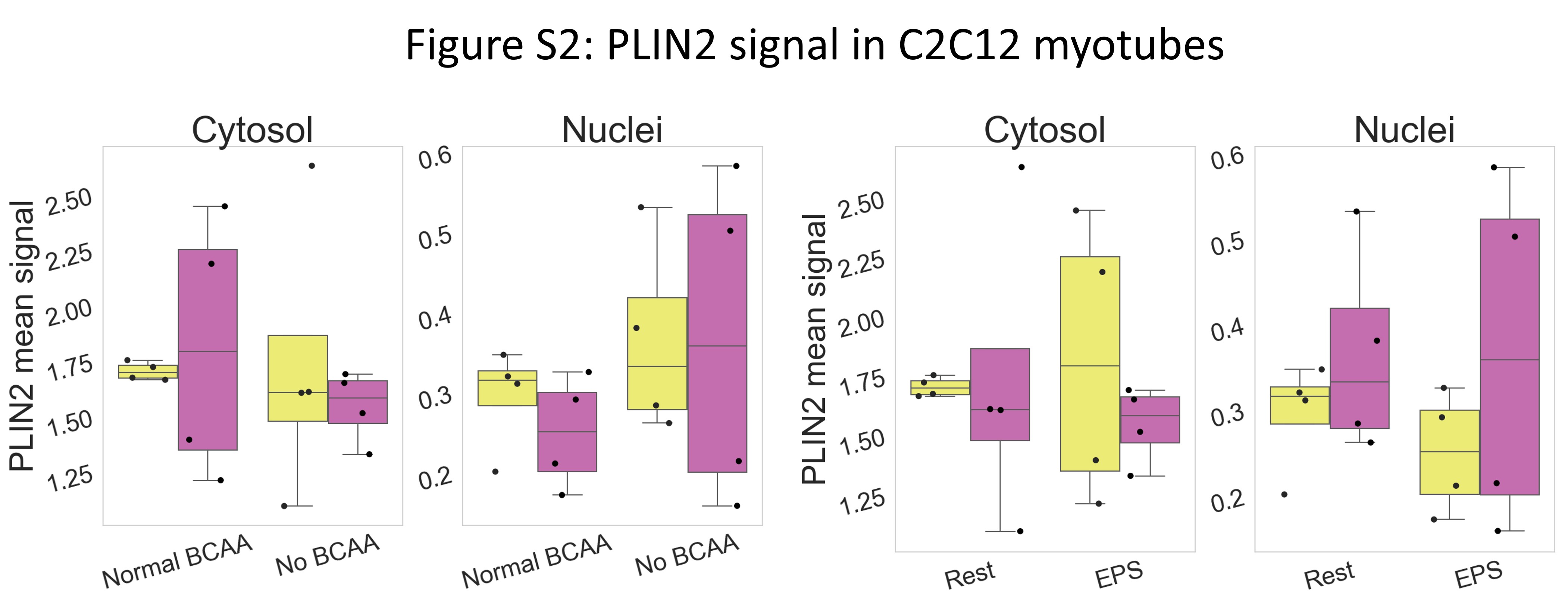

Supplement: Supplementary file 1 [file ijms-24-04282-s001.zip › supp_Updated/Sup2.jpg]

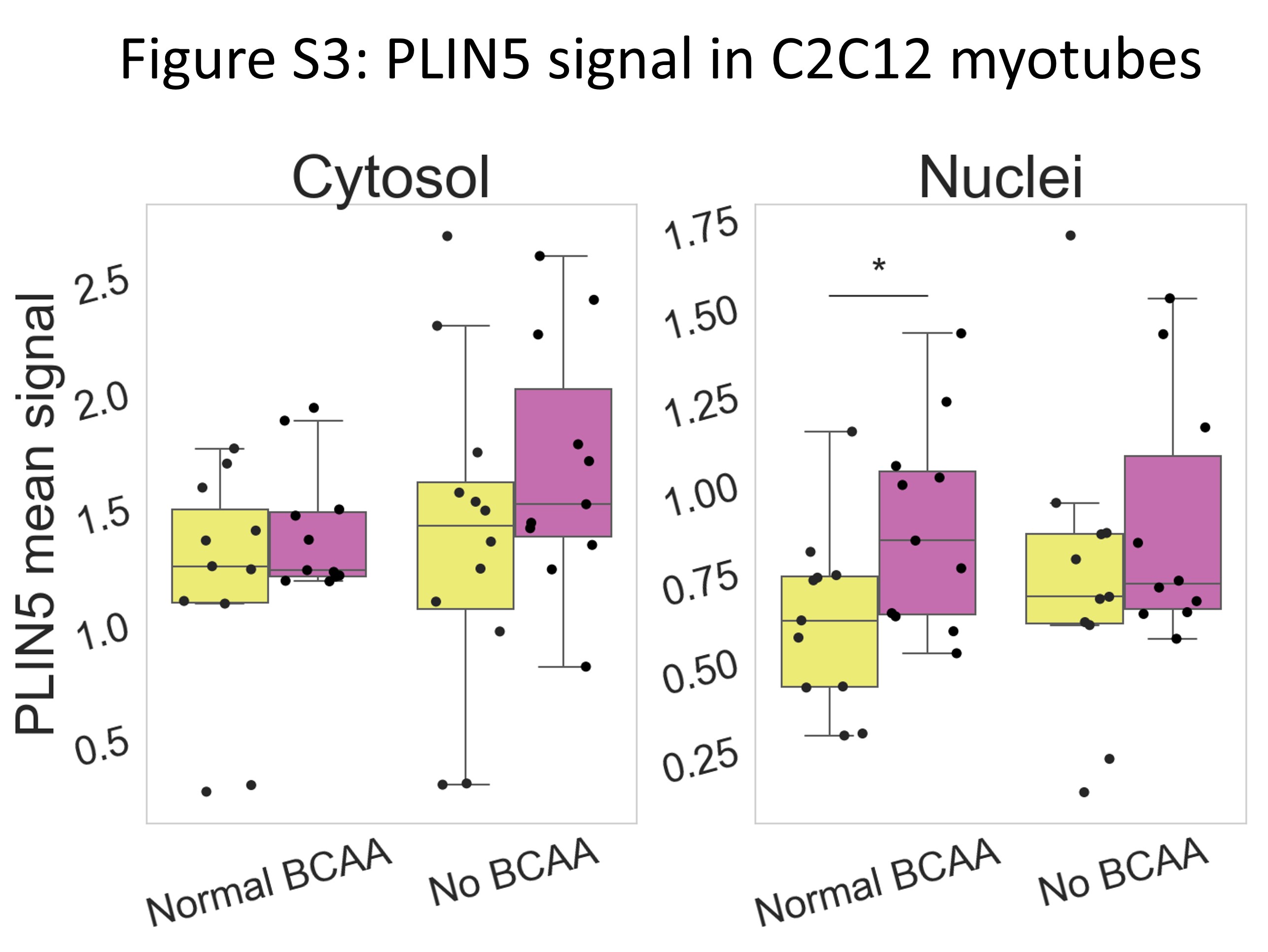

Supplement: Supplementary file 1 [file ijms-24-04282-s001.zip › supp_Updated/Sup3.jpg]

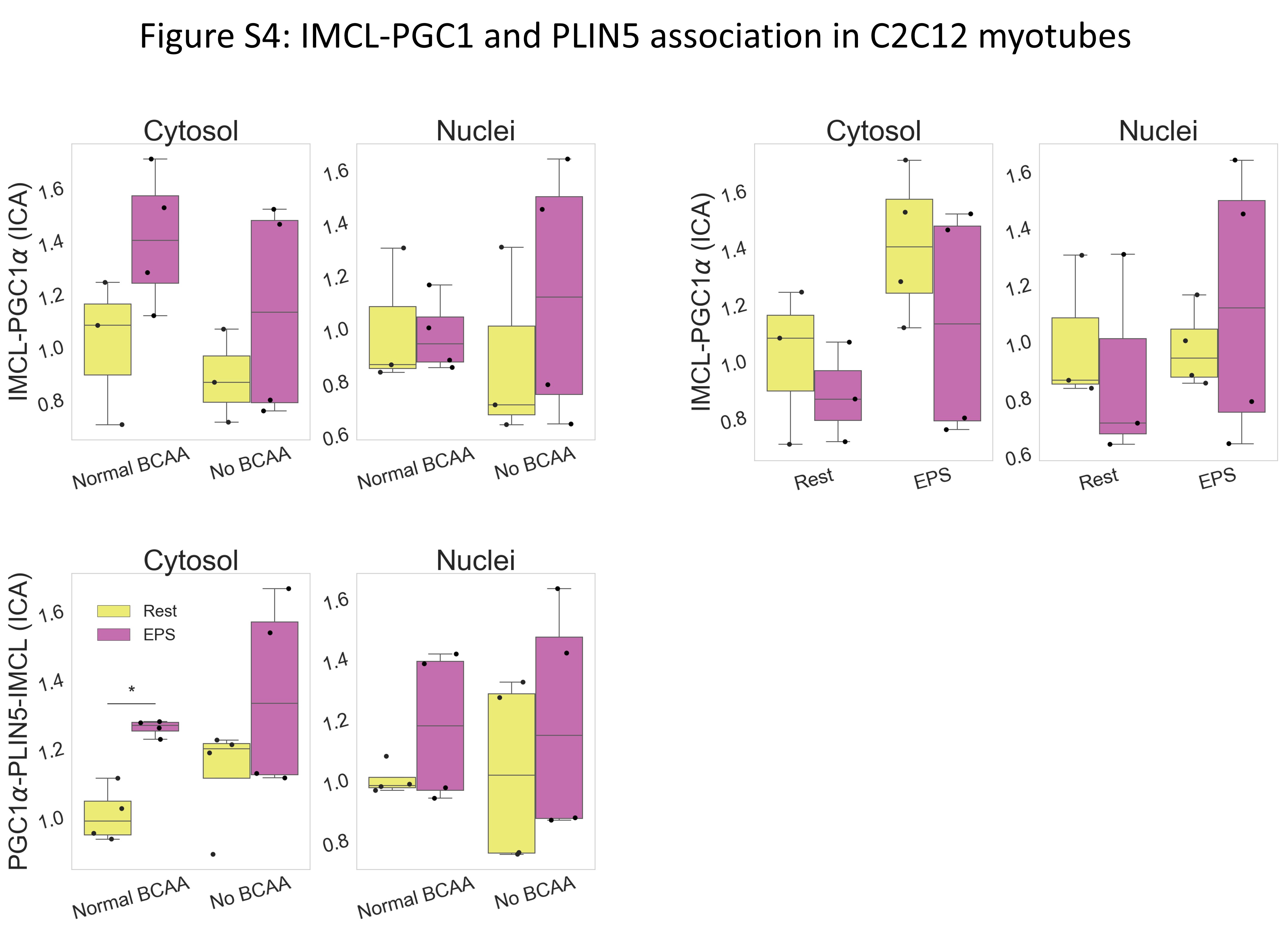

Supplement: Supplementary file 1 [file ijms-24-04282-s001.zip › supp_Updated/Sup4.jpg]

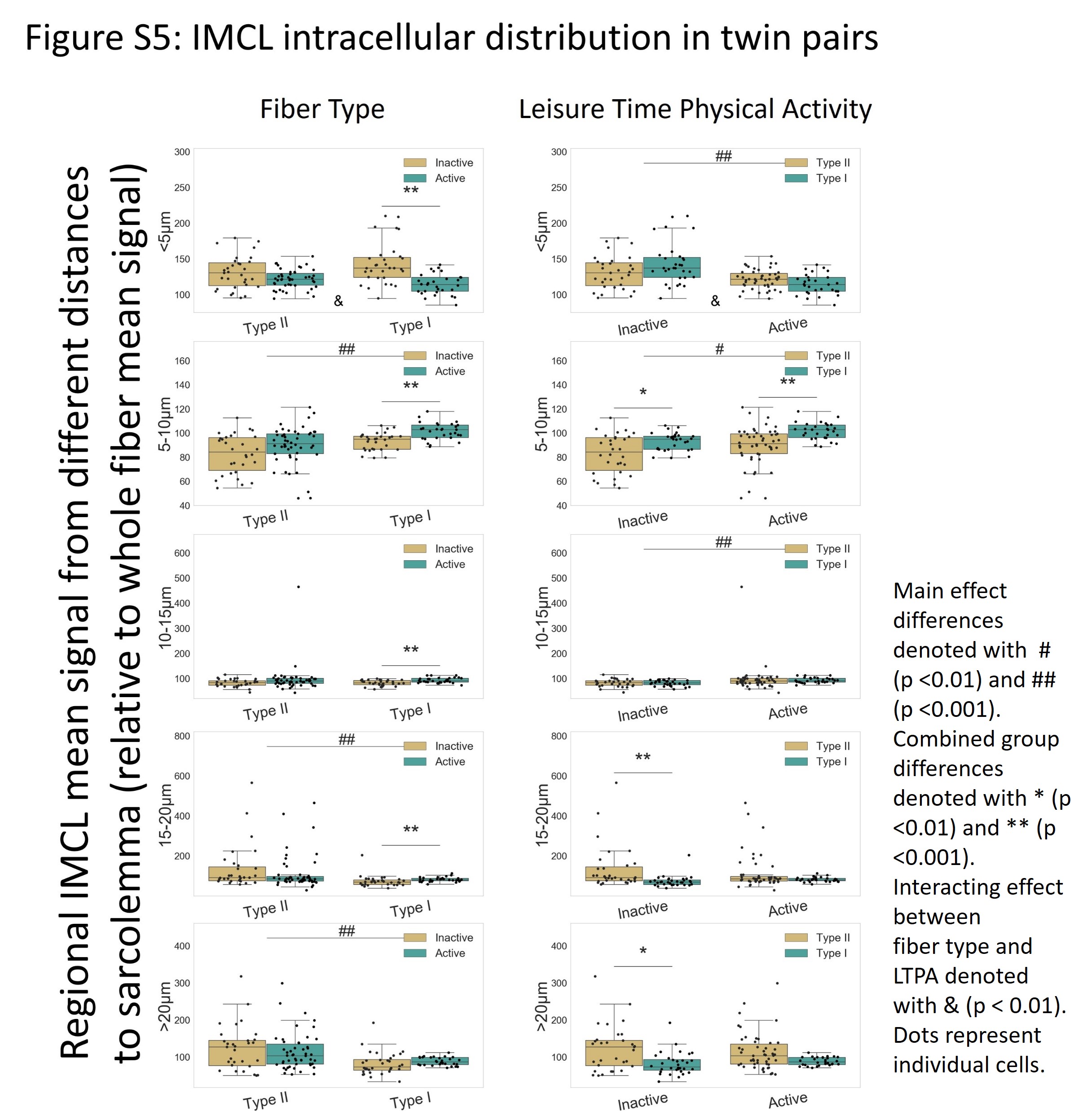

Supplement: Supplementary file 1 [file ijms-24-04282-s001.zip › supp_Updated/Sup5.jpg]

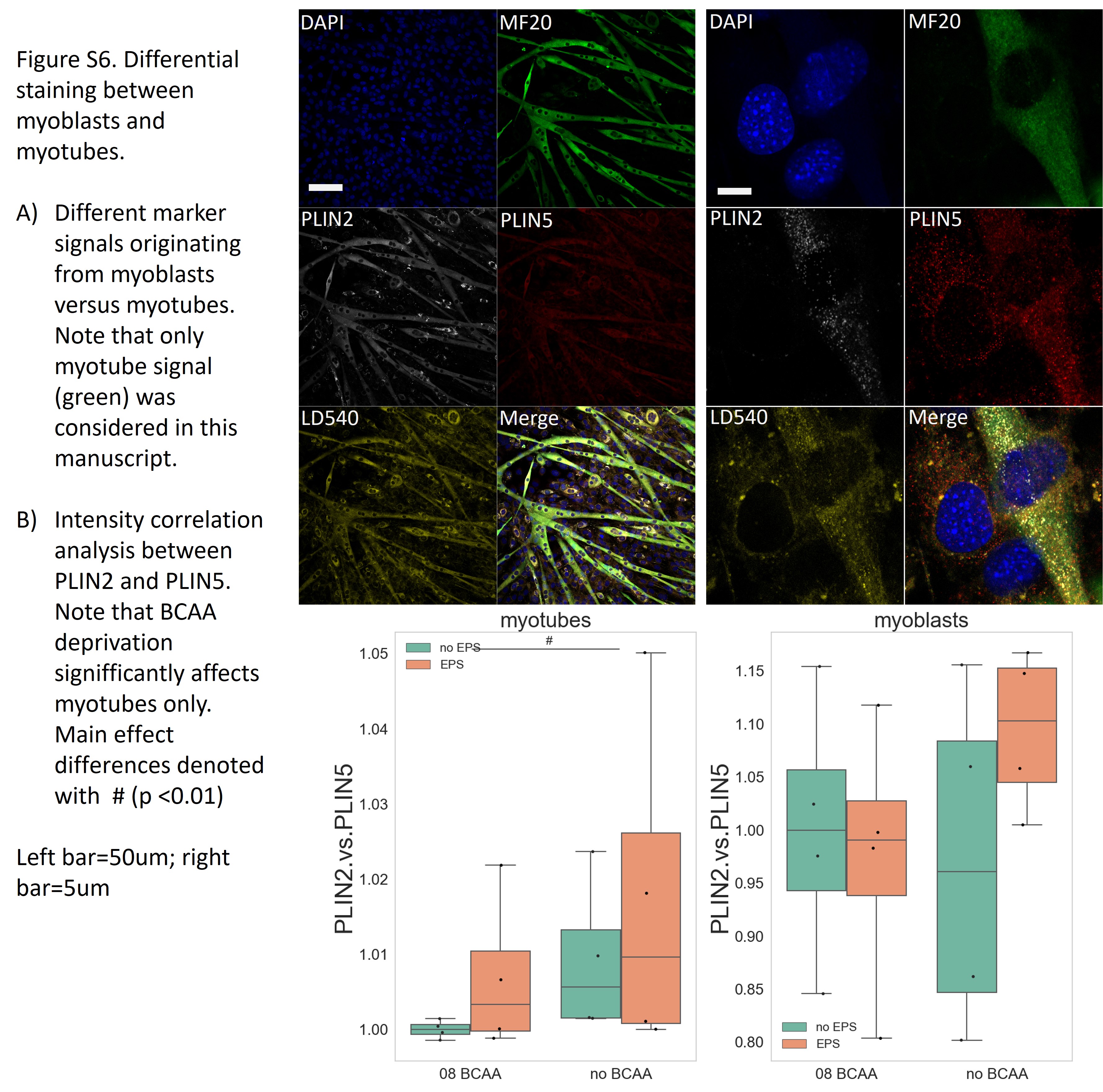

Supplement: Supplementary file 1 [file ijms-24-04282-s001.zip › supp_Updated/Sup6.jpg]

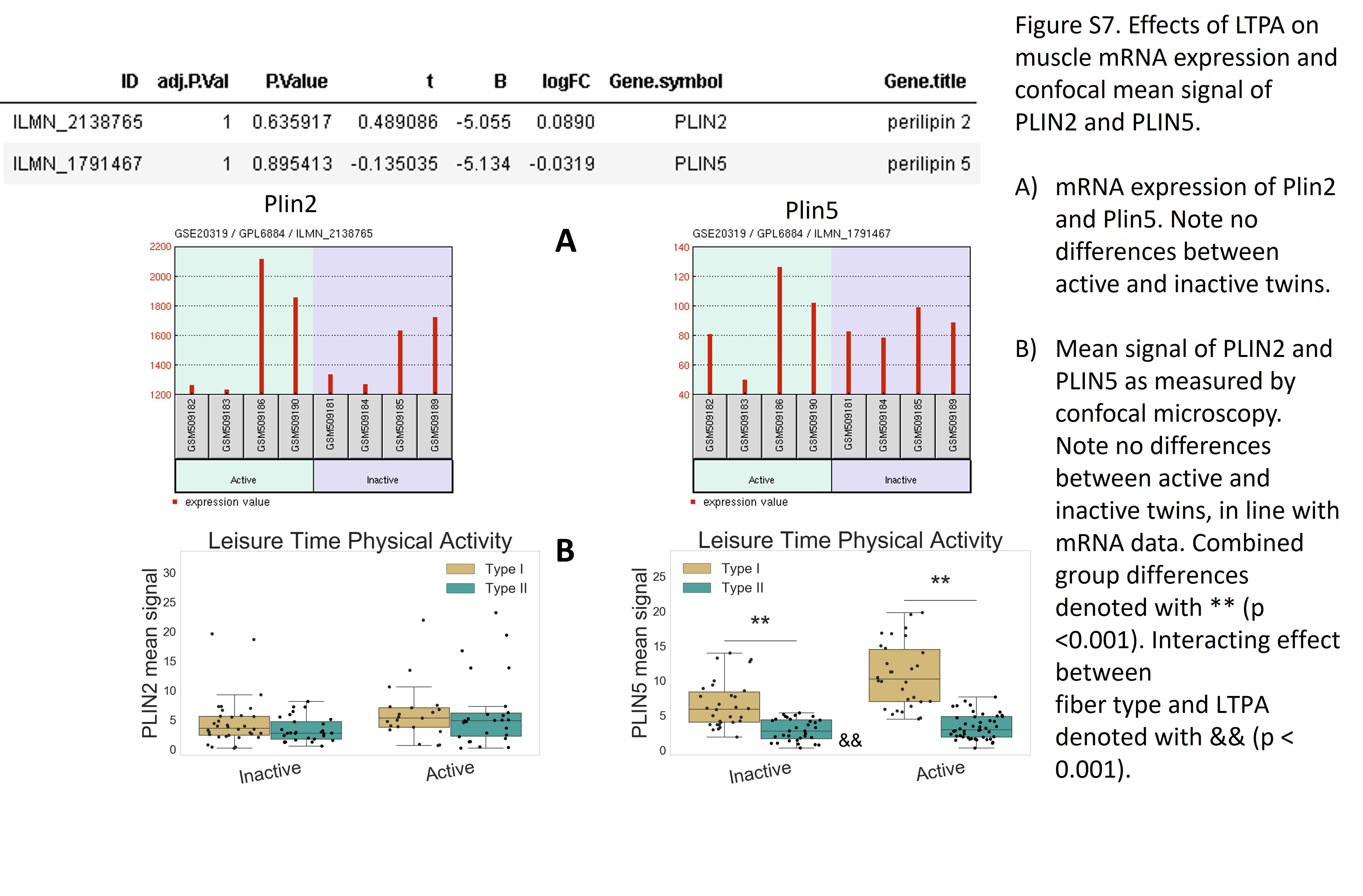

Supplement: Supplementary file 1 [file ijms-24-04282-s001.zip › supp_Updated/Sup7.jpg]

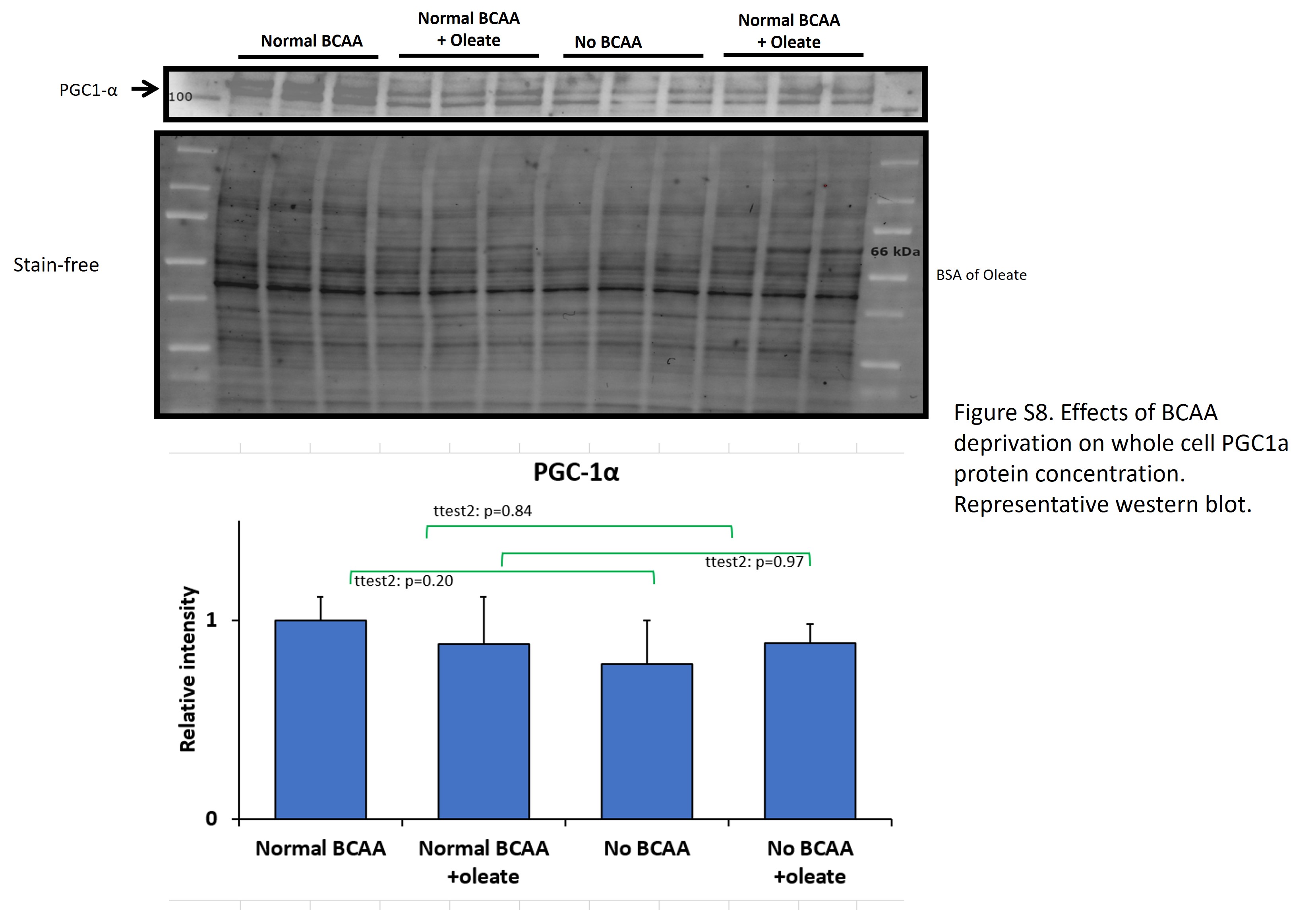

Supplement: Supplementary file 1 [file ijms-24-04282-s001.zip › supp_Updated/Sup8.jpg]
